# Essential Elements and Isoflavonoids in the Prevention of Prostate Cancer

**DOI:** 10.3390/nu14061225

**Published:** 2022-03-14

**Authors:** Iwona J. Stanisławska, Ramona Figat, Anna K. Kiss, Barbara Bobrowska-Korczak

**Affiliations:** 1 Department of Bromatology, Medical University of Warsaw, Banacha 1, 02-097 Warsaw, Poland; iwona.stanislawska@wum.edu.pl; 2 Department of Environmental Health Sciences, Medical University of Warsaw, Banacha 1, 02-097 Warsaw, Poland; ramona.figat@wum.edu.pl; 3 Department of Pharmacognosy and Molecular Basis of Phytotherapy, Medical University of Warsaw, Banacha 1, 02-097 Warsaw, Poland; anna.kiss@wum.edu.pl

**Keywords:** isoflavonoid, anti-genotoxicity, prostate cancer, chemoprevention, zinc, selenate, copper, iron, calcium

## Abstract

The intake of selected minerals, especially zinc, calcium and selenium, and high consumption of dietary isoflavones are recognised as factors influencing prostate cancer risk. Moreover, changes in levels of some essential elements are characteristic of the disease. Here, we examined the combined effects of main dietary isoflavonoids (genistein, daidzein and its metabolite, equol) and minerals implicated in prostate cancer, namely zinc, selenium, copper, iron and calcium, on LNCaP prostate cancer cells proliferation. Secondly, we evaluated the influence of the combinations on genotoxicity of model mutagens, 4-nitroquinoline oxide (4NQO) and 2-aminoanthracene (2AA), in the *umu* test. All combinations of isoflavonoids and minerals inhibited prostate cancer cells growth. However, only mixtures with iron ions had significantly stronger effect than the phytochemicals. Interestingly, we observed that only genistein attenuated genotoxicity of 4NQO. The addition of any tested mineral abolished this effect. All tested isoflavonoids had anti-genotoxic activity against 2AA, which was significantly enhanced in the presence of copper sulphate. Our results indicate that the tested minerals in physiological concentrations had minimal influence on the anti-proliferative activity of isoflavonoids. However, they significantly modulated the anti-genotoxic effects of isoflavonoids against both metabolically activated and direct mutagens. Thus, the minerals intake and nutritional status may modulate protective action of isoflavonoids.

## 1. Introduction

Prostate cancer is the second most commonly diagnosed cancer and the fifth cause of cancer mortality in men worldwide. The GLOBOCAN database estimates 1.41 million new cases and 375,300 deaths from prostate cancer in 2020 [1]. Prostate cancer incidence and mortality rates, though rising, remain lowest in most Asian regions. Although the highest mortality rates are observed in countries with a significant population of African descent, in regions with the highest incidence rate, i.e., Australia and New Zealand, Northern and Western Europe and Northern America, prostate cancer is also a prominent cause of cancer death [2,3]. Distinct ethnic and geographic patterns of symptomatic disease incidence combined with lesser prevalence diversity of latent prostate cancer as well as studies on migrant populations brought attention to the influence of modifiable factors on prostate cancer risk [4,5,6]. A substantial number of them depend on dietary habits. The intake or nutritional status of selected nutrients as well as consumption of specific food products is linked with modulation of prostate cancer risk [5].

Soy food products, a major source of isoflavone phytoestrogens characteristic for traditional East and Southeast Asia cuisine, are among the dietary factors inversely associated with prostate cancer risk [5,7,8]. Soy isoflavones were shown to influence prostate cancer cells through numerous mechanisms in in vitro and in vivo studies and impact their proliferation and viability (reviewed in [9]). Moreover, soy isoflavones, especially genistein, exerted anti-genotoxic effects against direct and promutagens [10]. Some essential minerals were also indicated as factors influencing prostate health. High consumption of dairy and calcium is linked with increased risk of prostate cancer, whereas selenium intake is associated inversely with it [5]. Zinc is an element with a profound role in prostate gland function and pathology [11]. The results of epidemiological studies are often not conclusive [5] or even outright contradictory, as seen for calcium [12,13,14]. Moreover, changes in levels of several essential elements in serum and prostate tissue are associated with prostate cancer and may correlate with severity of the disease. Decreased levels of zinc and selenium as well as an increase in copper, iron and calcium levels are among the characteristics seen in prostate cancer patients [15,16,17]. However, it is difficult to determine whether changes in essential minerals levels are causal factors or markers/results of pathological process [13]. A direct effect of minerals level on prostate cancer cells was demonstrated in in vitro models for zinc [18,19,20], selenium [21,22,23], copper [24,25], iron [26] and calcium [27,28]. Some studies reported unfavourable results of minerals supplementation in culture medium [28,29]. Moreover, modulation of the genotoxic effect of (pro)mutagens by minerals is a described phenomenon [30]. Exposure of prostate cancer cells to minerals is natural in an in vivo setting. The influence of isoflavonoids depends on individual dietary habits and use of supplements [8]. Thus, the possibility of mutual modulation of the effects of isoflavonoids and minerals exists. However, to our knowledge such data are scarce and available to some extent only for mixtures of isoflavonoids and selenium compounds [31,32,33,34].

In this study, we evaluated the anti-proliferative, genotoxic and anti-genotoxic potential of dominant dietary isoflavones (genistein, daidzein and its microbiota metabolite equol) in combinations with selected minerals, and which tissue levels were implicated in prostate cancer development and progression. Significant modulation of anti-genotoxic effects of isoflavonoids by the tested minerals suggests that chemopreventive potential of dietary isoflavonoids may be modified by minerals balance or nutritional status. We used the LNCaP prostate cancer cell line, which is characterized by relative susceptibility to anti-proliferative effects of isoflavonoids in comparison to androgen-independent cell lines (e.g., DU-145, PC-3) [35,36,37,38,39], as model suitable for detection of both enhanced and attenuated effects of tested combinations.

## 2. Materials and Methods

### 2.1. Materials

LNCaP human prostate cancer cell line was obtained from ATCC (Manassas, VA, USA). *Salmonella typhimurium* strain TA1535/pSK1002 was purchased from Deutsche Sammlung von Mikroorganismen und Zellkulturen GmbH (DSMZ, Inhoffenstraße 7B 38124 Braunschweig, Germany). DMEM medium supplemented with phenol red, Ultraglutamine and high glucose (4.5 g/L) was from Lonza (Verviers, Belgium). The solutions of penicillin/streptomycin, 0.25% trypsin-EDTA and phosphate buffered saline (PBS) were from Gibco (Paisley, UK). Genistein (≥98%, HPLC), daidzein (≥98%), equol (≥99%, TLC), fetal bovine serum (FBS), sodium pyruvate solution (100 mM), deoxyribonucleic acid sodium salt type XIV: from herring testes, bisbenzimide H 33258, camptothecin, 4-nitroquinoline N-oxide (4NQO), 2-aminoanthracene (2AA), d-glucose 6-phosphate disodium salt hydrate (G-6-P) and 2-nitrophenyl β-d-galactopyranoside (ONPG) were purchased from Sigma-Aldrich (Poznań, Poland). Nicotinamide adenine dinucleotide phosphate (NADP) was purchased from MP Biomedicals. Zinc sulphate (ZnSO_4_ × 7H_2_O, ≥99.5%), sodium selenate (Na_2_SeO_4_, ≥98%), copper(II)/cupric sulphate (CuSO_4_ × 5H_2_O, ≥99%), iron(II) sulphate (FeSO_4_ × 7H_2_O, ≥99%), DMSO were obtained from POCH (Gliwice, Poland) and calcium chloride (CaCl_2_ × 6H_2_O, ≥98%) was from Chempur (Piekary Śląskie, Poland). Methanol was purchased from Merck (Warsaw, Poland).

#### The Solutions Preparation

Stock solutions of isoflavones (genistein daidzein and equol), camptothecin, 4NQO and 2AA were prepared in sterile DMSO in aseptic conditions. The salts of tested elements (Zn, Se, Cu, Fe, Ca) were dissolved in deionised water (Merck Milipore, Darmstadt, Germany). The working solutions of elements were prepared by their dilution with 0.9% saline and filtration through 0.22 µM filter in aseptic conditions.

### 2.2. Assessment of the Effect on Human Prostate Cancer Cells Proliferation and Iability/Apoptosis

#### 2.2.1. Cell Culture

LNCaP cells (passage 12–18) were maintained in DMEM medium supplemented with 10% (*v*/*v*) FBS, sodium pyruvate (1 mM), penicillin (100 U/mL) and streptomycin (100 μg/mL) at 37 °C in 5% CO_2_ humidified atmosphere.

#### 2.2.2. Proliferation Studies

LNCaP cells (4–5 × 10^4^/well) were seeded in 24-well plates and allowed to attach for 48 h and treated for the indicated time with Zn (0.5 μg/mL/7.6 μM), Se (30 ng/mL/0.38 μM), Cu (0.5 μg/mL/7.9 μM), Fe (1.5 μg/mL/26.9 μM), Ca (40 μg/mL/1 mM), 10 μM isoflavone (genistein, daidzein or equol) and the combinations of isoflavone with each element. The final concentration of DMSO was 0.05%. It must be noted that DMEM contains 1.8 mM of calcium. Thus, control cells were exposed to 1.8 mM (72 μg/mL) of the mineral, and Ca-treated cells to total concentration of 2.8 mM (112 μg/mL) calcium. The cells were then gently washed with 0.9% saline and lysed by brief sonication in deionised water. The cell proliferation was quantified by measurement of bisbenzimide H 33,258–DNA complexes fluorescence on Synergy 4 microplate reader with Gen5 software (BioTek Instruments Inc., 100 Tigan St, Winooski, VT 05404, USA) [40].

#### 2.2.3. Fluorescent Imaging

LNCaP cells, treated with Se, Fe and genistein for 48 h, were stained for 1 h with Hoechst 33,258 dye added directly to the culture medium (final concentration 5 μg/mL). The images were recorded with the DS-Fi1 camera connected with Eclipse TS100F inverted microscope with Epi-Fluorescent attachment (DAPI filter) and NIS-Elements BR 2.3 software.

### 2.3. Anti-Genotoxicity Assessment

#### 2.3.1. Umu-Test

The *umu*-test detects the induction of bacterial DNA-damage response, the SOS system, in the strain *S. typhimurium* TA1535/pSK1002. The test strain carries pSK1002 plasmid, which contains the fusion of *umuC*, encoding one of SOS response proteins, and *lacZ* genes. The expression of β-galactosidase protein is fully controlled by *umuC* gene. Thus, β-galactosidase activity illustrates the level of SOS response and the genotoxic potency of the tested compound [41,42].

In the present study, the *umu*-test was performed in the micro-plate format according to the ISO guideline [43]. The β-galactosidase activity was determined by the degree of ONPG cleavage measured as sample absorbance at 420 nm. The extent of SOS system activation was presented as induction ratio (IR), calculated as the ratio of β-galactosidase activity in the presence of the tested compound(s) to that of negative control. An IR value equal or greater than 1.5 indicated the genotoxic effect of the tested compound(s). The optical density at 600 nm was used to describe the bacteria growth. A growth ratio value higher than 0.5 relative to negative control indicated lack of growth inhibition. Both measurements were performed with Asys UVM340 Hightech microplate spectrophotometer.

#### 2.3.2. Metabolic Activation

The S9 fraction from rat liver was used for metabolic activation of the tested compounds and the model mutagen. The male Sprague-Dawley rats were administered single dose of Aroclor 1254 (500 mg/kg b.w. in soybean oil) five days before S9 fraction isolation. The P450 cytochrome level in the isolate was evaluated and the aliquots were stored at −80 °C. The S9 reaction mix was prepared as described by Maron and Ames [44].

#### 2.3.3. Determination of Anti-Genotoxicity by the Umu Test

In order to the establish anti-genotoxic potential of tested compounds, their effects on the action of two model genotoxic agents, 4NQO without the metabolic activation with rat liver S9 fraction and 2AA with metabolic activation, were evaluated. Firstly, the genotoxic potentials of 4NQO (0.05 and 0.25 μg/mL) and 2AA (1 and 10 μg/mL) were measured by the *umu*-test. Simultaneously, the genotoxicity of the tested isoflavones (10 μM), minerals (0.5 μg/mL Zn, 30 ng/mL Se, 0.5 μg/mL Cu, 1.5 μg/mL Fe, 40 μg/mL Ca) and their combinations was investigated. Subsequently, the modulation of 4NQO and 2AA genotoxicity by the isoflavones, the elements and their mixtures in previously used concentrations was investigated. The rate of anti-genotoxicity (%) of the tested sample was calculated as the inhibition of IR induced by the genotoxic agent (4NQO or 2AA) at the particular concentration according to the following formula:

$$\mathrm{Anti}-\mathrm{genotoxicity}\%=\left(1-\frac{{\mathrm{IR}}_{\mathrm{genotoxin}+\mathrm{sample}}}{{\mathrm{IR}}_{\mathrm{genotoxin}}}\right)\times 100\%.$$

### 2.4. Statistical Analysis

The data were obtained from at least three independent experiments performed in triplicate. Statistical significance was established using ANOVA and Dunnett’s and Tuckey’s post hoc tests. *p* values < 0.05 were considered significant. Data are presented as mean ± standard deviation (SD).

## 3. Results

### 3.1. Proliferation of LNCaP Cells

**Figure 1:**
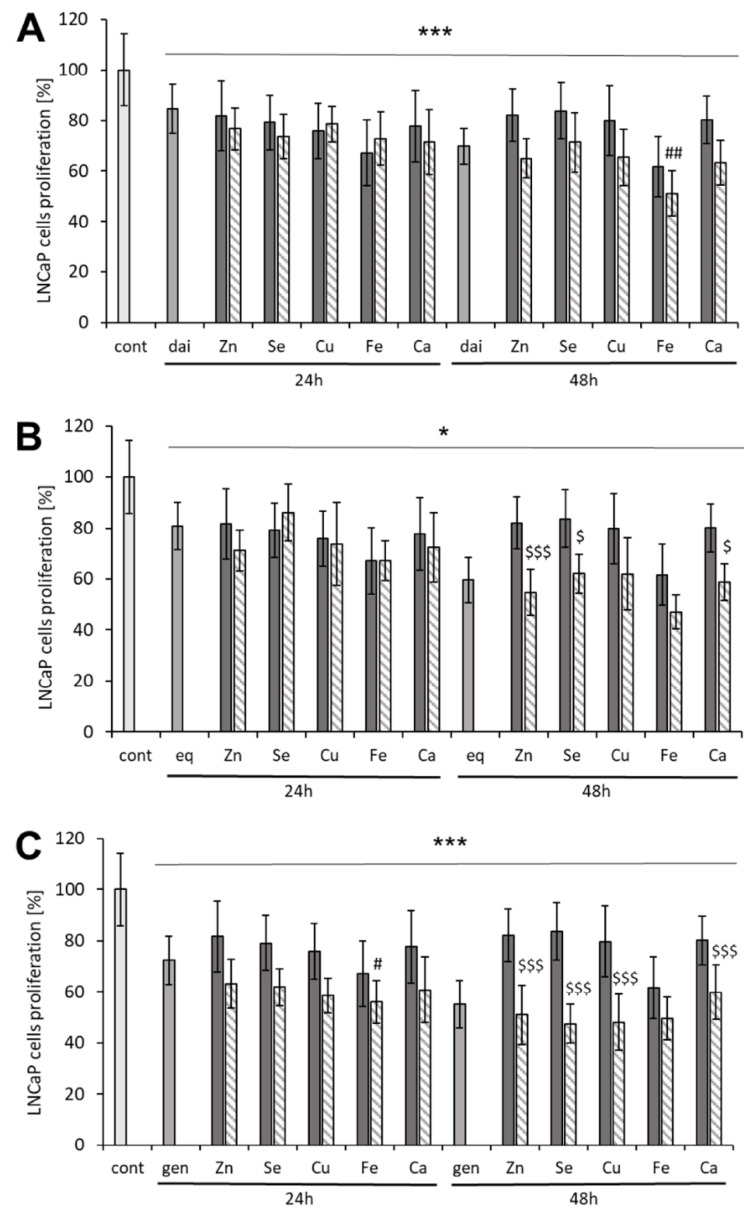
The anti-proliferative effects of (micro)elements and isoflavones on LNCaP cells. The solid bars represent the effects of single agents and the patterned bars the effects of combinations of (**A**) daidzein (dai), (**B**) equol (eq) and (**C**) genistein (gen) with designated elements. * *p* < 0.05, *** *p* < 0.001 vs. control; # *p* < 0.05, ## *p* < 0.01 vs. 10 μM isoflavone; $ *p* < 0.05, $$$ *p* < 0.001 vs. respective element.

The supplementation of each mineral in culture medium moderately decreased LNCaP prostate cancer cells proliferation to 67–81% and 62–84% of control after 24 h and 48 h of incubation, respectively (*p* < 0.001) (Figure 1). In this in vitro assay, Fe had the most pronounced effect (38 ± 3% inhibition after 48 h), which was greater than those of other minerals (*p* < 0.001). There was no significant change in the minerals’ anti-proliferative effect with the extension of exposure time from 24 h to 48 h.

The isoflavonoids had similar anti-proliferative effect at 10 μM (16–28% of inhibition) after 24 h of incubation to the minerals. Contrary to the effect of the minerals, extension of incubation time to 48 h led to further decrease of proliferation by daidzein (84 ± 10% vs. 70 ± 7%, *p* < 0.01), equol (81 ± 9% vs. 60 ± 9%, *p* < 0.001) and genistein (72 ± 9 vs. 55 ± 9%, *p* = 0.001). Equol and genistein acted significantly stronger than Zn (82 ± 10%), Se (84 ± 11%), Cu (80 ± 14%) and Ca (80 ± 9%) after 48 h of incubation (*p* < 0.001).

The effects of the combinations of minerals with isoflavonoids were largely comparable to those of the respective isoflavonoids. The mixtures with Fe were the only ones which had improved anti-proliferative activity in comparison with daidzein (Figure 1A) and genistein (Figure 1C). The combination of Fe with equol showed a tendency to act stronger than the isoflavone metabolite (*p* ≤ 0.09, Figure 1B). However, no additive or synergistic effect was observed as effects of Fe and the Fe combinations with isoflavonoids did not differ significantly. Our observations indicate that the effects of tested isoflavonoid–mineral combinations were comparable to the effect of the more active constituent of given combination. We have seen no attenuation of the anti-proliferative effect.

### 3.2. Viability/Apoptosis of LNCaP Cells

**Figure 2:**
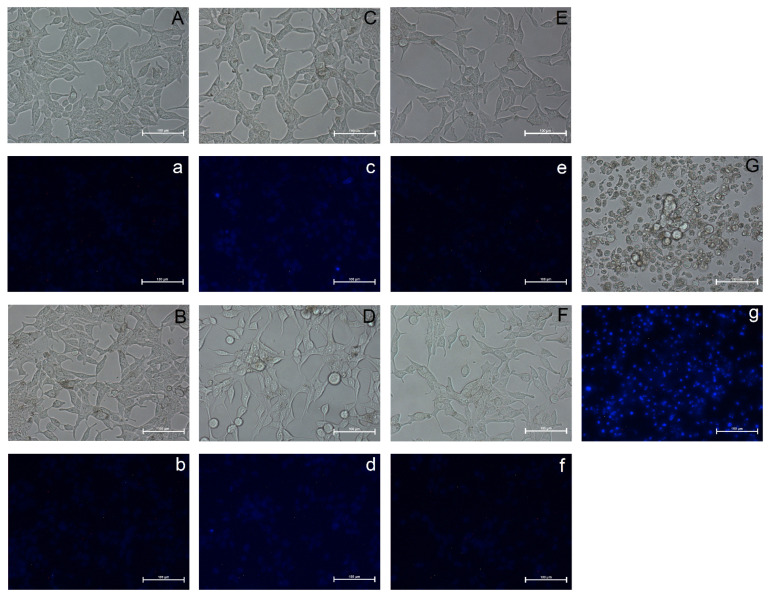
The effect of genistein, selenium and iron on LNCaP cells viability. The representative photographs of LNCaP cells: control (**A**,**a**), treated with genistein (**B**,**b**), selenium (**C**,**c**), iron (**E**,**e**) and combinations of genistein with Se (**D**,**d**) and Fe (**F**,**f**). The cells treated with 2 µM camptothecin (**G**,**g**) were positive control. Scale bar–100 µm. Photographs marked with uppercase letters present cells morphology. Photographs marked with lowercase letters show the fluorescence of Hoechst 33,258 stained cells.

We evaluated the effect of the most active isoflavone and mineral in the proliferation test, i.e., genistein and Fe, on the viability and apoptosis of LNCaP cells. The action of Se was also assessed based on significant associations of selenium levels and prostate cancer occurrence. As shown in Figure 2, genistein, Fe or Se used individually did not induce apoptosis in LNCaP cells. Neither the spindle shape nor the morphology of the cells were changed (Figure 2B,C,E) in comparison to control cells (Figure 2A). We did not observe increased amount of cells with condensed chromatin or apoptotic nuclei which was dyed bright blue with Hoechst stain in cells as seen for positive control—the camptothecin-treated cells (Figure 2g). Similarly, no such changes were observed in cells treated with a mixture of genistein and Fe (Figure 2F,f). However, some less adhered, rounded cells (Figure 2D) without marks of apoptosis (Figure 2d) were observed after treatment with combination of Se and genistein. Cells treated with genistein, minerals (Se, Fe) or their combinations show lightly dyed nuclei with uncondensed chromatin.

### 3.3. Anti-Genotoxicity Assessment

Neither the tested isoflavones nor the minerals showed genotoxic activity in the *umu* test (IR < 1.5) or inhibited the growth of bacteria (G > 0.5), irrespective of the metabolic activation (Table 1).

Genotoxicity of direct mutagen, 4-nitroquinoline oxide (4NQO), was significantly inhibited by genistein (20%), calcium (18%) and ferrous (20%) ions (Table 2), although only when a higher concentration (0.25 mg/L) of 4NQO was used. However, addition of any tested minerals, even those showing anti-genotoxic effect, abolished protective activity of genistein. Other isoflavonoids and minerals, either individually or in combination, had no effect on 4NQO genotoxicity (Table 2 and Table 3, graphical summary Table 4).

In the experiments with 2-aminoanthracen (2AA), a premutagen requiring metabolic activation by enzymes of S9 liver fraction, a significant decrease in its genotoxicity by genistein (by 17–32%), equol (29–34%) and daidzein (19%) was observed (Table 5 and Table 6). Genistein and equol had stronger anti-genotoxic effect against a lower concentration (1 mg/L) of 2AA, while daidzein activity was observed solely against higher concentration (5 mg/L) of 2AA. Ions of copper and iron were also able to attenuate 2AA genotoxicity by 26–33% and 14–21%, respectively (Table 5). The effects of both ions were more pronounced for a lesser concentration of premutagen.

Introduction of minerals modulated anti-genotoxic effects of isoflavonoids (graphic summary Table 7). Copper ions enhanced the effect of genistein (significance reached against 5 mg/L 2AA). Conversely, mixtures of genistein with zinc and selenate ions had attenuated anti-genotoxic effect in comparison to the isoflavone, especially against a lower concentration of 1 mg/L 2AA (Table 5).

The mixtures of daidzein with copper ions and, to a lesser extent, iron ions had significantly stronger anti-genotoxic activity against 2AA in comparison to daidzein. Although no statistical significance was reached, addition of zinc, calcium and selenate seemed to attenuate the action of daidzein (Table 6).

The mixtures of equol with copper and iron ions were more effective against 2AA genotoxicity than the isoflavonoid. The combination of equol with calcium ions had a contrary effect. Presence of zinc or selenate ions did not modulate the action of equol (Table 6).

It is worth noting that the effects of the mixtures containing copper ion were stronger than the effects of the respective isoflavone and copper ions used separately. The strongest anti-genotoxic effect (51% inhibition) was observed for the mixture of equol and copper ions. None of the tested isoflavones and minerals, either individually or in combination, increased the genotoxicity of model mutagens 4NQO and 2AA. Similarly, none of the used compounds or their mixtures were toxic to the bacteria (G > 0.5; Table 1, Table 2, Table 3, Table 4, Table 5, Table 6 and Table 7).

## 4. Discussion

In this study, we examined the combined effects of selected essential elements (Zn, Se, Cu, Fe and Ca) and major dietary isoflavones—genistein, daidzein and its gut microbiota metabolite equol on hormone-dependent LNCaP prostate cancer cells and potency of model mutagens. The chosen concentrations of minerals were within the physiological range observed in human serum [12,15] and tissues [16,17]. Used concentrations of isoflavonoids corresponded to the maximal levels observed in prostatic tissue after soy isoflavones oral supplementation in doses achievable with high consumption of soy products [45].

We observed decreased proliferation in LNCaP cells in the presence of 10 μM tested isoflavones in accordance with the results of earlier studies [35,36,37,46]. Relative anti-proliferative activity of the isoflavones was also consistent with the reported pattern (genistein ≥ equol > daidzein) [46,47,48]. Genistein (10 μM) did not induce LNCaP cells apoptosis as described previously [9,37].

The supplementation of cell culture medium with tested minerals resulted only in a moderate decrease in cell proliferation. The tested minerals (Fe and Se) also did not induce apoptosis of prostate cancer cells. Although proapoptotic activity and decreased viability was described for iron and selenium compounds, it was observed at higher concentrations than used in the present study and, in the case of selenium, concerned selenite not selenate ions [21,22,26]. With the exception of iron, addition of essential elements did not significantly influence the effects of isoflavones. It is noteworthy that none of the isoflavone–mineral combinations had attenuated anti-proliferative effect. However, none of them showed stronger effect than the more active constituent of given mixture. With few exceptions such as selenium, the data on interactions between essential elements and isoflavones concerning prostate cancer are scarce.

Previous studies reported that inorganic zinc had strong inhibitory effect on LNCaP cells (IC_50_ = 100 ng/mL), induced apoptosis [18,19] and decreased PSA activity and LNCaP invasiveness [49]. Here the effects of zinc ions on growth of LNCaP cells were weaker, similarly to the recent work reporting increased degradation of AR protein in zinc-treated cells [20]. This discrepancy may be explained by differences in experimental design. The presence of serum in culture medium was a probable factor as it might modulate availability of zinc for the cells [50]. Both isoflavones and inorganic zinc are known to inhibit androgen receptor [20,51,52,53] and NF-κB signalling pathways [54,55]. There is evidence that several polyphenols modulate zinc distribution in prostatic cells [56]. Interaction with inorganic zinc was shown to sensitise cells to growth inhibition/cytotoxicity caused by epigallocatechin gallate [57] and paclitaxel [55]. No enhancement of anti-proliferative effect was observed for combinations of zinc and isoflavones in our study nor, to our knowledge, described in literature. However, isoflavones influenced zinc levels and expression of zinc transporters in prostate tissue in vivo [58].

Supplementation of inorganic selenium compounds in culture medium inhibited growth of prostate cancer cells [21,22]. Induction of apoptosis was also observed, albeit at higher concentrations than used in the present study (i.e., ≈380 nM) [22,23]. Contrarily, low concentrations of selenium, in form of selenite, were reported to stimulate repair of oxidative damage [59] and even LNCaP cells growth [60]. The combinations of genistein and selenite were previously shown to have enhanced growth inhibitory and pro-apoptotic effect on androgen dependent and independent prostate cancer cells [31,32]. Such phenomena were not observed in present study probably due to use of selenate (Se +6) rather than selenite (Se +4) and lower concentration of the compound in the mixture. Selenate had a significantly attenuated effect on prostate cancer cell growth and viability in comparison to selenite [21]. However, a few studies described a decrease in factors associated with prostate cancer risk in laboratory animals upon isoflavone and selenium supplementation [33,34].

Inorganic copper was noted to reduce prostate cancer cells (LNCaP and PC-3) proliferation at concentrations close to that used in the present study in accordance with our observations [24]. In another study, higher copper concentrations (≥100 μM) decreased the viability of PC-3 cells, but in the selected concentration range were shown to promote viability of HPV-immortalized RWPE-1 cells [25]. Copper ions were able to potentiate anti-cancer activity of compounds able to form copper chelates [61] and/or modulate oxidative stress in cells [24,62]. Similarly, iron ions (200 µM) decreased proliferation and viability of prostate cancer cells, albeit ferric ions were reported to have lower activity than that of ferrous ions seen in present study, i.e., the decrease did not reach significance for concentrations 10–100 μM [26]. It is worth noting that iron supplementation enhanced anti-cancer effects of clinically used anti-androgen, bicalutamide, through exacerbation of oxidative stress in vitro and in vivo [26]. However, it is a delicate balance as lower concentrations of ferric ions (100 µM) were seen to increase the invasiveness of prostate cancer cells in a redox-dependent mechanism [29].

Here, we did not observe significant potentiation of the anti-proliferative effect of isoflavones combinations with copper or iron ions in comparison to the effects of both mixture components used individually. As described in the works cited above, oxidative stress is important mechanism of copper and iron cytotoxicity. Metal ions interact with flavonoids, including isoflavones, forming chelates and/or changing oxidation state [63], which may contribute to the modulation of their biological activity [56,64], e.g., epigallocatechin gallate with copper and zinc ions [24,57]. In contrast to some other flavonoids, isoflavones are able to reduce cupric to cuprous ions but not ferric to ferrous ions. Moreover, isoflavones are rather weak chelators among flavonoids and taking into account relative concentrations used in this study formation of both iron and copper chelates with isoflavones seems unlikely [63,65].

Calcium is a macromineral with vital role in cells function and its altered signalling, through various mechanisms, is implicated in prostate cancer [66]. The effects of extracellular calcium levels are not clear [5]. It must be noted that in serum calcium levels are under tight homeostatic control [67]. Epidemiological studies report that higher serum calcium levels are associated with increased [12], unchanged [13] or decreased [14,68] prostate cancer risk. Elevated concentrations of extracellular calcium promoted growth of prostate cancer cells derived from bone metastases [27,28]. The prostate cells not derived from bone metastases did not react to increased extracellular calcium levels from 0.5 (indicative of severe hypocalcaemia in vivo) to 2.5 mM [28]. We observed a slight but significant decrease in proliferation in LNCaP cells derived from lymph node metastases in response to an increase in calcium concentration from 1.8 to 2.8 mM.

The prevention of DNA changes caused by chemical, physical or biologic factors is among main mechanisms of chemoprevention [69]. Thus, we explored the effects of isoflavones, minerals and their combinations on activity of model mutagens, direct genotoxin 4NQO and promutagen 2AA. As expected, isoflavones did not show genotoxicity and exerted protective effect against direct (only genistein) and indirect mutagens in an *umu* test [10,70,71,72]. Isoflavonoids are able to inhibit numerous microsomal enzymes [73], which may to some extent explain their protective effect against metabolically activated promutagen. Anti-genotoxicity assessment showed consistent enhancement of isoflavonoids activity against 2AA-induced mutagenesis in the presence of cupric ions. Such effect was not seen in tests against 4NQO, where metal ions abolished protective effects of genistein. Copper ions demonstrated ability to increase mutagenicity of 4NQO and to reduce mutagenicity of S9 activated 2AA in *S. typhimurium* revertant test [30], albeit at much higher concentration than used here. Although there was report describing increased radical scavenging activity of genstein-cupric ion complex [74], such interaction with daidzein and equol is impossible due to lack of 4-oxo–5-hydroxy chelating site [63]. Thus, the effect observed here may be caused by modulation of isoflavonoids interaction with S9 fraction enzymes in the presence of cupric ion.

Prostate cancer is characterised by a long latency period preceding occurrence of symptomatic disease, which makes it a suitable target for preventative interventions, e.g., through dietary means. Use of isoflavonoids and minerals supplements is popular in Western societies [75,76,77]. Our preliminary results tentatively indicate that combining selected minerals, e.g., copper with isoflavonoids may offer some increased protection against environmental promutagens. Combining achievable in vivo concentrations of isoflavonoids and selected minerals (Zn, Se, Cu, Fe, Ca) did not result in direct enhancement of activity against hormone-dependent prostate cancer cells. However, protection against the effects of environmental threats such as (pro)mutagens is an important aspect of chemoprevention [69]. Thus, significant modulation of isoflavonoids anti-genotoxic activity by the minerals indicates that minerals nutritional status and metabolic balance may be among the factors influencing individual protective potential of isoflavonoid exposure.

## 5. Conclusions

In conclusion, the current study shows that the tested minerals in physiological concentrations have minimal influence on the anti-proliferative activity of isoflavonoids. However, they significantly modulate the anti-genotoxic effects of isoflavonoids against both metabolically activated and direct mutagens. Thus, the minerals intake and nutritional status may modulate protective action of isoflavonoids.

## Figures and Tables

**Table 1:** Genotoxicity assessment—the values of growth (G) and induction (IR) ratios for the tested compounds and their combinations with (+S9) and without metabolic activation (−S9).

Tested Mixture	+S9		−S9	
	G (Mean ± SD)	IR (Mean ± SD)	G (Mean ± SD)	IR (Mean ± SD)
Negative Control	1.00 ± 0.07	1.00 ± 0.13	1.00 ± 0.06	1.00 ± 0.13
gen	1.16 ± 0.17	0.78 ± 0.16	1.28 ± 0.35	0.75 ± 0.15
Zn	1.04 ± 0.05	0.99 ± 0.16	1.09 ± 0.13	1.02 ± 0.24
Cu	1.07 ± 0.11	0.93 ± 0.11	1.09 ± 0.12	0.87 ± 0.17
Ca	1.09 ± 0.15	0.99 ± 0.12	1.11 ± 0.09	0.93 ± 0.19
Se	1.09 ± 0.13	0.98 ± 0.15	1.14 ± 0.11	0.92 ± 0.19
Fe	0.98 ± 0.10	0.97 ± 0.10	1.10 ± 0.10	0.90 ± 0.19
gen + Zn	1.03 ± 0.15	1.03 ± 0.21	1.18 ± 0.42	0.80 ± 0.23
gen + Cu	0.99 ± 0.11	1.03 ± 0.19	0.88 ± 0.09	1.01 ± 0.21
gen + Ca	1.03 ± 0.08	1.02 ± 0.19	1.13 ± 0.14	0.86 ± 0.13
gen + Se	1.04 ± 0.11	1.05 ± 0.14	1.00 ± 0.10	1.03 ± 0.24
gen + Fe	1.06 ± 0.07	1.03 ± 0.13	1.03 ± 0.09	1.04 ± 0.16
dai	0.94 ± 0.12	0.98 ± 0.13	1.25 ± 0.28	0.86 ± 0.14
dai + Zn	0.92 ± 0.10	1.06 ± 0.18	1.05 ± 0.08	1.03 ± 0.17
dai + Cu	1.00 ± 0.13	1.10 ± 0.13	1.11 ± 0.15	1.02 ± 0.17
dai + Ca	1.02 ± 0.12	0.95 ± 0.11	1.12 ± 0.15	0.97 ± 0.10
dai + Se	1.06 ± 0.07	1.02 ± 0.07	1.14 ± 0.11	1.03 ± 0.12
dai + Fe	1.03 ± 0.08	0.99 ± 0.08	1.07 ± 0.11	0.87 ± 0.16
eq	1.06 ± 0.09	0.84 ± 0.10	1.18 ± 0.33	0.83 ± 0.16
eq + Zn	1.04 ± 0.10	0.91 ± 0.11	0.89 ± 0.14	1.24 ± 0.33
eq + Cu	1.10 ± 0.07	0.90 ± 0.12	0.99 ± 0.14	1.31 ± 0.52
eq + Ca	1.10 ± 0.10	0.92 ± 0.13	0.98 ± 0.07	1.04 ± 0.18
eq + Se	1.14 ± 0.10	0.98 ± 0.14	1.07 ± 0.07	1.23 ± 0.30
eq + Fe	1.05 ± 0.07	0.93 ± 0.13	1.02 ± 0.08	1.15 ± 0.50

Each value is expressed as mean ± standard deviation from at least 3 independent biological evaluation.

**Table 2:** Evaluation of anti-genotoxic activity based on G and IR values for the genotoxin—4NQO (4-nitroquinoline oxide) (0.25 mg/L, 0.05 mg/L)—and the mixture of NQO with genistein, minerals and their combinations.

Tested Mixture	G (Mean ± SD)	IR (Mean ± SD)	%Anti-Genotox.
Negative Control	1.00 ± 0.06	1.00 ± 0.13	
NQO 0.25 mg/L	0.95 ± 0.11	6.06 ± 1.48	
gen	1.12 ± 0.24	**4.84 ± 1.47**	**20%**
Zn	1.01 ± 0.11	5.20 ± 1.44	
Cu	1.00 ± 0.12	5.14 ± 1.41	
Ca	1.11 ± 0.05	**4.94 ± 1.28**	**18%**
Se	1.11 ± 0.05	5.19 ± 1.63	
Fe	1.08 ± 0.10	**4.86 ± 1.27**	**20%**
gen + Zn	0.98 ± 0.18	5.95 ± 1.24 ^a^	
gen + Cu	0.85 ± 0.09	6.26 ± 0.84 ^a^	
gen + Ca	1.01 ± 0.09	5.52 ± 0.58 ^a^	
gen + Se	0.95 ± 0.09	5.98 ± 0.89 ^a^	
gen + Fe	0.99 ± 0.07	5.50 ± 0.51 ^a^	
NQO 0.05 mg/L	0.99 ± 0.10	2.64 ± 0.63	
gen	1.01 ± 0.12	2.48 ± 0.58	
Zn	1.01 ± 0.11	2.36 ± 0.46	
Cu	0.98 ± 0.10	2.42 ± 0.61	
Ca	1.13 ± 0.08	2.22 ± 0.54	
Se	1.10 ± 0.09	2.39 ± 0.48	
Fe	1.10 ± 0.10	2.24 ± 0.52	
gen + Zn	0.89 ± 0.11	2.77 ± 0.78	
gen + Cu	0.78 ± 0.06	3.04 ± 0.79	
gen + Ca	1.00 ± 0.11	2.55 ± 0.45	
gen + Se	0.97 ± 0.07	2.74 ± 0.53	
gen + Fe	1.04 ± 0.09	2.56 ± 0.34	

Each value is expressed as mean ± standard deviation from at least 3 independent biological evaluations. Means that are significant different from positive control (2AA) are bolded (*p* < 0.05). ^a^ Significantly different from the effect of genistein (10 μM) without minerals. %anti-genotox. of the tested samples is calculated as the inhibition of IR induced by genotoxin (4NQO).

**Table 3:** Evaluation of anti-genotoxic activity based on G and IR values for the genotoxin—4NQO (0.25 mg/L, 0.05 mg/L)—and the mixture of NQO with daidzein, equol and the combinations with minerals.

Tested Mixture	G (Mean ± SD)	IR (Mean ± SD)	%Anti-Genotox.
Negative Control	1.00 ± 0.09	1.00 ± 0.16	
NQO 0.25 mg/L	0.78 ± 0.12	9.92 ± 3.06	
Dai	1.03 ± 0.25	7.92 ± 2.64	
dai + Zn	0.93 ± 0.10	8.57 ± 2.50	
dai + Cu	0.99 ± 0.14	9.23 ± 2.76	
dai + Ca	1.06 ± 0.21	8.60 ± 2.87	
dai + Se	1.09 ± 0.20	8.67 ± 2.57	
dai + Fe	1.04 ± 0.20	8.06 ± 2.47	-
Eq	0.97 ± 0.26	9.25 ± 4.77	
eq + Zn	0.80 ± 0.16	10.49 ± 3.87	
eq + Cu	0.84 ± 0.11	9.67 ± 2.86	
eq + Ca	0.90 ± 0.19	9.64 ± 3.62	
eq + Se	0.96 ± 0.15	9.36 ± 2.56	
eq + Fe	0.90 ± 0.14	8.37 ± 2.58	
NQO 0.05 mg/L	0.97 ± 0.15	2.80 ± 1.56	
Dai	0.94 ± 0.16	2.48 ± 1.65	
dai + Zn	0.86 ± 0.10	2.78 ± 1.87	
dai + Cu	0.92 ± 0.14	2.70 ± 1.53	
dai + Ca	1.06 ± 0.18	2.54 ± 1.37	
dai + Se	1.12 ± 0.15	2.54 ± 1.36	
dai + Fe	1.09 ± 0.16	2.36 ± 1.11	-
Eq	1.06 ± 0.27	2.75 ± 1.63	
eq + Zn	0.89 ± 0.13	3.25 ± 2.03	
eq + Cu	0.96 ± 0.11	3.07 ± 1.49	
eq + Ca	1.04 ± 0.08	2.82 ± 1.39	
eq + Se	1.03 ± 0.11	3.17 ± 1.73	
eq + Fe	0.95 ± 0.10	2.80 ± 1.37	

Each value is expressed as mean ± standard deviation from at least 3 independent biological evaluations.

**Table 4:** Summary of anti-genotoxic activity of isoflavonoids, minerals and their combinations against direct mutagen 4NQO (0.25 mg/L; 0.05 mg/L).

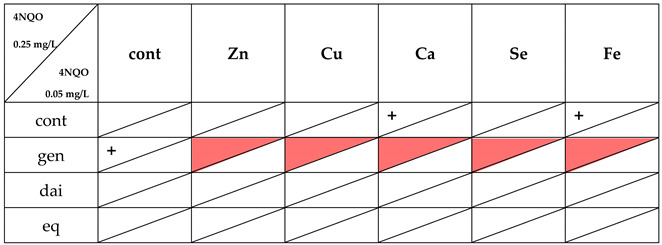

+—anti-genotoxicty of used compound(s) ≤ 20%. Red colour—significant attenuation of anti-genotoxic activity of isoflavonoid (*p* > 0.05); gen—genistein; dai—daidzein; eq—equol.

**Table 5:** Evaluation of anti-genotoxic activity based on G and IR values for the genotoxin—2AA (5 mg/L, 1 mg/L)—and the mixture of 2AA with genistein, minerals and combinations.

Tested Mixture	G (Mean ± SD)	IR (Mean ± SD)	%Anti-Genotox.
Negative Control	1.00 ± 0.07	1.00 ± 0.13	
2AA 5 mg/L	1.02 ± 0.08	2.17 ± 0.39	
Gen	1.21 ± 0.17	**1.79 ± 0.25**	**17%**
Zn	1.08 ± 0.05	2.20 ± 0.31	
Cu	1.16 ± 0.14	**1.60 ± 0.25**	**26%**
Ca	1.19 ± 0.13	2.36 ± 0.36	
Se	1.17 ± 0.12	2.11 ± 0.40	
Fe	1.07 ± 0.07	**1.87 ± 0.27**	**14%**
gen + Zn	0.98 ± 0.09	2.06 ± 0.42	
gen + Cu	0.98 ± 0.13	**1.48 ± 0.34 ^a^**	**32%**
gen + Ca	1.07 ± 0.13	2.02 ± 0.58	
gen + Se	1.09 ± 0.16	1.96 ± 0.55	
gen + Fe	1.09 ± 0.11	**1.69 ± 0.28**	**22%**
2AA 1 mg/L	1.00 ± 0.07	2.00 ± 0.53	
Gen	1.16 ± 0.17	**1.35 ± 0.23**	**32%**
Zn	1.03 ± 0.08	1.77 ± 0.42	
Cu	1.11 ± 0.12	**1.34 ± 0.30**	**33%**
Ca	1.16 ± 0.10	1.86 ± 0.44	
Se	1.18 ± 0.10	1.79 ± 0.40	
Fe	1.10 ± 0.06	**1.59 ± 0.27**	**21%**
gen + Zn	0.92 ± 0.08	1.89 ± 0.51 ^a^	
gen + Cu	0.95 ± 0.13	**1.26 ± 0.21**	**37%**
gen + Ca	1.08 ± 0.14	**1.58 ± 0.29**	**21%**
gen + Se	1.04 ± 0.18	**1.61 ± 0.34 ^a^**	**19%**
gen + Fe	1.04 ± 0.13	**1.44 ± 0.32**	**28%**

Each value is expressed as mean ± standard deviation from at least 3 independent biological evaluations. Means that are significant different from positive control (2AA) are bolded (*p* < 0.05). ^a^ Significantly different from the effect of genistein (10 μM) without minerals. %anti-genotox. of the tested samples is calculated as the inhibition of IR induced by genotoxin (2AA).

**Table 6:** Evaluation of anti-genotoxic activity based on G and IR values for the genotoxin–2AA (5 mg/L, 1 mg/L)—and the mixture of 2AA with daidzein, equol and the combinations with minerals.

**Tested Mixture**	**G** **(Mean ± SD)**	**IR** **(Mean ± SD)**	**%Anti-Genotox.**
Negative Control	1.00 ± 0.08	1.00 ± 0.10	
2AA 5 mg/L	0.86 ± 0.09	2.91 ± 0.54	
dai	0.99 ± 0.13	**2.36 ± 0.45**	**19%**
dai + Zn	0.89 ± 0.14	2.56 ± 0.78	
dai + Cu	0.94 ± 0.15	**1.71 ± 0.28 ^a^**	**41%**
dai + Ca	0.98 ± 0.17	2.81 ± 0.66	
dai + Se	1.03 ± 0.15	2.49 ± 0.47	
dai + Fe	1.00 ± 0.12	**2.03 ± 0.36**	**30%**
eq	1.03 ± 0.09	**2.08 ± 0.30**	**29%**
eq + Zn	0.96 ± 0.06	**2.19 ± 0.38**	**25%**
eq + Cu	1.01 ± 0.06	**1.61 ± 0.32 ^b^**	**45%**
eq + Ca	1.04 ± 0.12	**2.55 ± 0.50 ^b^**	
eq + Se	1.08 ± 0.13	2.22 ± 0.40	**24%**
eq + Fe	0.96 ± 0.13	**1.80 ± 0.24 ^b^**	**38%**
2AA 1 mg/L	0.92 ± 0.10	2.37 ± 0.48	
dai	0.92 ± 0.16	2.09 ± 0.45	
dai + Zn	0.88 ± 0.09	**1.93 ± 0.25**	**19%**
dai + Cu	0.94 ± 0.14	**1.54 ± 0.22 ^a^**	**35%**
dai + Ca	0.99 ± 0.13	2.15 ± 0.32	
dai + Se	1.00 ± 0.15	2.00 ± 0.31	
dai + Fe	0.90 ± 0.15	**1.75 ± 0.29 ^a^**	**26%**
eq	0.99 ± 0.11	**1.58 ± 0.18**	**34%**
eq + Zn	0.94 ± 0.10	**1.62 ± 0.17**	**32%**
eq + Cu	1.03 ± 0.09	**1.17 ± 0.14 ^b^**	**51%**
eq + Ca	1.09 ± 0.10	**1.76 ± 0.23 ^b^**	**26%**
eq + Se	1.16 ± 0.10	**1.80 ± 0.38**	**24%**
eq + Fe	1.06 ± 0.06	**1.51 ± 0.26**	**36%**

Each value is expressed as mean ± standard deviation from at least 3 independent biological evaluations. Means that are significant different from positive control (2AA) are bolded (*p* > 0.05). ^a^ Significantly different from the effect of daidzein (10 μM) without minerals. ^b^ Significantly different from the effect of equol (10 μM) without minerals. %anti-genotox. of the tested samples is calculated as the inhibition of IR induced by genotoxin (2AA).

**Table 7:** Summary of anti-genotoxic activity of isoflavonoids, minerals and their combinations against metabolically activated promutagen 2AA (5 mg/L; 1 mg/L).

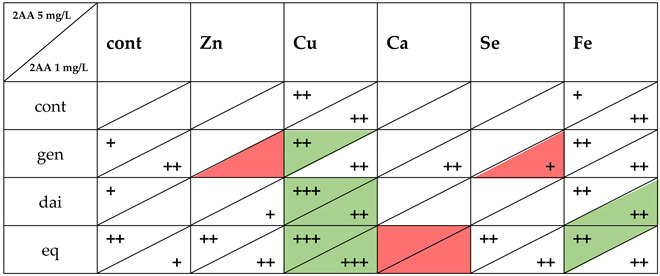

+—anti-genotoxicty of used compound(s) ≤ 20%; ++—anti-genotoxicty of used compound(s) 21–40%; +++—anti-genotoxicty of used compound(s) ≥ 40%; Green colour—mixtures showing significantly stronger anti-genotoxic activity in comparison to used isoflavonoid (*p* > 0.05). Red colour—mixtures showing significantly lower anti-genotoxic activity in comparison to used isoflavonoid (*p* > 0.05); gen—genistein; dai—daidzein; eq—equol.

## Data Availability

Not applicable.
